# The cysteine-reactive small molecule ebselen facilitates effective SOD1 maturation

**DOI:** 10.1038/s41467-018-04114-x

**Published:** 2018-04-27

**Authors:** Michael J. Capper, Gareth S. A. Wright, Letizia Barbieri, Enrico Luchinat, Eleonora Mercatelli, Luke McAlary, Justin J. Yerbury, Paul M. O’Neill, Svetlana V. Antonyuk, Lucia Banci, S. Samar Hasnain

**Affiliations:** 1 Molecular Biophysics Group, Institute of Integrative Biology, Faculty of Health and Life Sciences, University of Liverpool, Liverpool, L69 7ZB UK; 2 Magnetic Resonance Centre (CERM), University of Florence, 50019 Sesto Fiorentino, Italy; 3 Interuniversity Consortium for Magnetic Resonance of Metallo Proteins (CIRMMP), 50019 Sesto Fiorentino, Italy; 4 Department of Biomedical, Clinical and Experimental Sciences, University of Florence, 50134 Florence, Italy; 5 Illawarra Health and Medical Research Institute, University of Wollongong, Wollongong, NSW 2522 Australia; 6 Department of Chemistry, University of Liverpool, Liverpool, L69 7ZD UK; 7 Department of Chemistry, University of Florence, 50019 Sesto Fiorentino, Florence Italy

## Abstract

Superoxide dismutase-1 (SOD1) mutants, including those with unaltered enzymatic activity, are known to cause amyotrophic lateral sclerosis (ALS). Several destabilizing factors contribute to pathogenicity including a reduced ability to complete the normal maturation process which comprises folding, metal cofactor acquisition, intra-subunit disulphide bond formation and dimerization. Immature SOD1 forms toxic oligomers and characteristic large insoluble aggregates within motor system cells. Here we report that the cysteine-reactive molecule ebselen efficiently confers the SOD1 intra-subunit disulphide and directs correct SOD1 folding, depopulating the globally unfolded precursor associated with aggregation and toxicity. Assisted formation of the unusual SOD1 cytosolic disulphide bond could have potential therapeutic applications. In less reducing environments, ebselen forms a selenylsulphide with Cys111 and restores the monomer–dimer equilibrium of A4V SOD1 to wild-type. Ebselen is therefore a potent bifunctional pharmacological chaperone for SOD1 that combines properties of the SOD1 chaperone hCCS and the recently licenced antioxidant drug, edaravone.

## Abstract (web summary)

Mutations in superoxide dismutase-1 (SOD1) cause amyotrophic lateral sclerosis (ALS). Here the authors present the SOD1 crystal structure bound to the small cysteine-reactive molecule ebselen and show that ebselen is a chaperone for SOD1.

## Introduction

Amyotrophic lateral sclerosis (ALS) is a fatal motor system neurodegenerative disease. A subset of ALS is caused by mutations to the gene encoding superoxide dismutase-1 (SOD1)^1^. Histopathology on tissues from humans and transgenic mutant SOD1 animal models has shown how SOD1 protein accumulates in Lewy-like bodies within the cytoplasm and mitochondria of motor neurons and support cells. These insoluble inclusions are replete with misfolded SOD1 which appears unmanageable by protein degradation pathways^2,3^.

Pioneering work on the catalytic activity of SOD1 and subsequent exploration of its structure proved that a range of post-translational modifications (PTMs) are necessary not only for its function, but more importantly with respect to ALS, for its stability^4^. While zinc binding and an intra-subunit disulphide are not directly involved in catalysis, they stabilize the molecule’s tertiary and quaternary structure^5–7^. ALS-related mutations reduce the ability of SOD1 to fold, bind necessary metal cofactors and predispose it to disulphide reduction, which in turn prevents its normal dimerization. As a result, a portion of cellular SOD1 is forced to reside in a destabilized or persistently misfolded state^8,9^. Exposure of normally internalized hydrophobic motifs leads to aberrant oligomerization, which manifests as disulphide-reduced, metal-free, insoluble SOD1 inclusions^10,11^. SOD1 is also observed to be disulphide reduced in people living with asthma. While this does not lead to aggregation, it does have harmful effects on the ability of SOD1 to perform its function^12,13^ and suggests that addressing the SOD1 disulphide reduction problem could be useful for the treatment of both diseases.

The propensity of SOD1 to unfold and aggregate has been shown to correlate with life expectancy after ALS symptom onset in humans and transgenic mouse models^8,14–16^. Thus, exogenous promotion of normal SOD1 maturation processes could divert nascent SOD1 away from the toxic pathway. Non-covalent ligand interactions have been demonstrated on the SOD1 β-barrel surface, but each failed to change the mutant thermal stability or inhibit aggregation in vitro possibly due to low binding affinity^17^. Conversely, platination of the solvent exposed and reactive Cys111 residues or covalently cross-linking them across the dimer interface groove can modulate disease-related characteristics. Both bismaleimidoethane (BMOE) and cisplatin increase SOD1 thermal stability, while the latter also inhibits in vitro and in-cell aggregation^18,19^. However, both interactions have been shown to inhibit heterodimer formation with the copper chaperone for SOD1 (hCCS), an important activator of SOD1^20^.

Ebselen is an organoselenium compound with broad antioxidant properties^21^. It is also an anti-inflammatory and has been tested as a treatment for a variety of conditions including stroke^22^, bipolar disorder^23^ and aneurysmal subarachnoid haemorrhage^24^. Interestingly, it has also been shown to reduce the cytotoxic burden of mutant SOD1 mitochondrial pathology^25^. Ebselen has strong pharmacological and structural similarities with the antioxidant edaravone, which very recently became only the second drug licensed for the treatment of ALS in the United States.

Here we demonstrate that ebselen and its analogue ebsulphur bind to SOD1 at the Cys111 site in vitro. This interaction shifts the SOD1 monomer–dimer equilibrium in favour of the dimer and returns the affinity of the A4V dimer formation to that of the wild-type. This occurs without covalent tethering of monomer subunits within the SOD1 dimer. Importantly the increased dimer stability does not prevent heterodimer formation with hCCS. This proof of principle is important as Cys111-targeted drug compounds could bind the free sulphydryl of both mutant or wild-type SOD1 and should not prevent structurally important hCCS-catalyzed activation. Furthermore, we show by in-cell and in vitro nuclear magnetic resonance (NMR) that ebselen reacts with disulphide-reduced SOD1 in the human cell cytosol causing the formation of the intra-subunit disulphide bond. In doing so, it promotes correct SOD1 folding and zinc binding. Directing the nascent protein down the correct PTM pathway prevents accumulation of unfolded species, thought to be the precursor of mutant SOD1 toxicity, premature degradation and aggregation^8,9^. Thus, ebselen rescues the toxic characteristics of mutant SOD1 through two independent disulphide formation and dimer stabilization mechanisms, deployment of which is dependent on the nature of the environment. While non-native disulphide bonding is often used as a strategy to stabilize recombinant proteins, here we describe a possible therapeutic application of stimulated disulphide bonding within human cells; an exciting possibility given the rarity of cytosolic disulphides and the specificity that could hardwire into the system. Ebselen’s on-target SOD1 pharmacological chaperone activity, in combination with its usage history and positive secondary pharmacology reminiscent of edaravone, indicates that it is a very promising lead not only in the search for therapeutics, which can modulate pathogenic SOD1 behaviour in ALS, but also asthma.

## Results

### Ebselen and ebsulphur form a covalent bond with SOD1 at Cys111

**Fig. 1:**
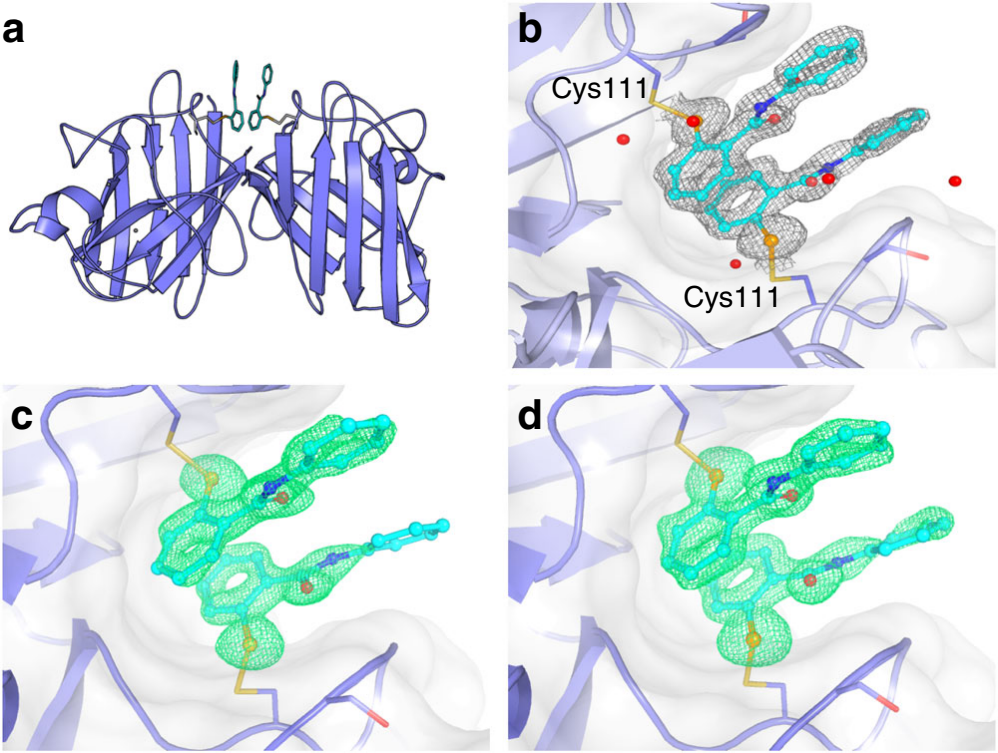
The structure of ebselen and ebsulphur bound to SOD1. **a** Cartoon representation of the SOD1 dimer with ebselen or ebsulphur molecules shown as ball and stick and bound to Cys111 residues at the dimer interface. **b** Electron density map (2Fo−Fc contoured at 1*σ*) showing the pose of two SOD1-bound ebselen molecules and proximal crystallographic waters. Electron density (maps mFo–DFc contoured at 3*σ*) of **c** ebsulphur and **d** ebselen showing details of binding at Cys111

Screening libraries of existing drugs, those that are no longer used or drugs that were not successful in the final stages of clinical trials, is an increasingly popular approach for therapeutic discovery. Drug repurposing offers a cheaper and faster route through early clinical trials as ADMET information is available, creating a major advantage for ‘orphan diseases’ such as ALS. This approach has resulted in historic as well as recent successes (see review^26^), including the licensing of edaravone in Japan and the US to slow ALS progression^27^. Given the susceptibility of Cys111 to oxidative modifications in SOD1-ALS and previous work on cisplatin and BMOE, we undertook a crystallographic screening of a small library of cysteine-reactive compounds known to have favourable properties (Supplementary Fig. 1). Ebselen and ebsulphur were found to form a covalent bond with SOD1 Cys111 (Fig. 1a, b) while derivatives with a benzyl substitution on the benzoisoselenazole or benzoisothiazolone nitrogen did not, possibly due to altered reactivity or steric hindrance in the dimer groove. Binding was confirmed using denaturing mass spectrometry (MS) (Supplementary Fig. 2).

The 1.3 and 1.5 Å resolution structures of wild-type, zinc metalated, disulphide intact SOD1 bound to ebsulphur and ebselen, respectively, allowed for the unambiguous placement of the compounds in the SOD1 dimer groove. They show the formation of a selenylsulphide or mixed disulphide 2.0 Å covalent bond, respectively, between ligand and Cys111 created by nucleophilic attack of the free Cys111 sulphydryl at the electrophilic Se–N and S–N centres contained within each respective heterocycle. Electron density is evident for both ligands except for a small part of one of the ebsulphur phenyl rings which projects into the surrounding solvent. This indicates some rotational freedom in this group (Fig. 1c and d). No ligand conjugation was observed at Cys6 and the intramolecular disulphide bond between Cys57 and Cys146 remains intact, as does the conformation of loop VI (Supplementary Fig. 3a and b). No large structural changes were seen between the amino acid backbone or side chains of ligand-conjugated SOD1 compared to unbound SOD1 crystallized under the same conditions (RMSD 0.24 Å, Supplementary Fig. 3c and d). Ebselen and ebsulphur form tight interactions within the SOD1 dimer groove, and a hydrogen bond is formed between the ligand carbonyl and a highly coordinated water molecule situated, on average, 2.55 Å away. Through this bond, the ligand is integrated into the water network forming an indirect link to the Gly108 backbone amide of one SOD1 monomer. This reflects the asymmetry of the ligand binding to each monomer. Ebselen and ebsulphur appear to form additional inter-dimer contacts through parallel stacking arrangement of their arylamine groups.

### Ebselen and ebsulphur potentiate SOD1 dimer formation

The propensity for mutant SOD1 to exist as a monomer is a key contributory factor in the pathology of SOD1-ALS. Two of the dimer interface mutants, A4V and I113T, including the most common and severe mutant, A4V, have been shown to exhibit significant dimer destabilization^28^. Indeed, preventing dimer dissociation has been shown to increase SOD1 thermal stability and inhibit aggregation in vitro^18,29^. Native MS preserves non-covalent interactions and allows quantification of tight homo-oligomer dissociation constants without reverting to chaotropic unfolding as well as the ability to resolve differentially modified protein species^16,30^. Native MS of the ligand-bound SOD1 showed the typical native MS profile of SOD1 where monomer arises as +7 and +8 multiply charged ions, and the dimer as +9 to +12 ions with the major component of dimer signal being 10+ and 11+ charged ions (Supplementary Figure 4). We observed no change in the charge state distribution with covalent attachment of ligands. After confirming similar MS profiles between unbound and ligand-bound SOD1, we determined the effect of ebselen and ebsulphur on the SOD1 monomer–dimer equilibrium by comparing the signal belonging to dimer and monomer across a range of concentrations (Supplementary Fig. 5).

**Fig. 2:**
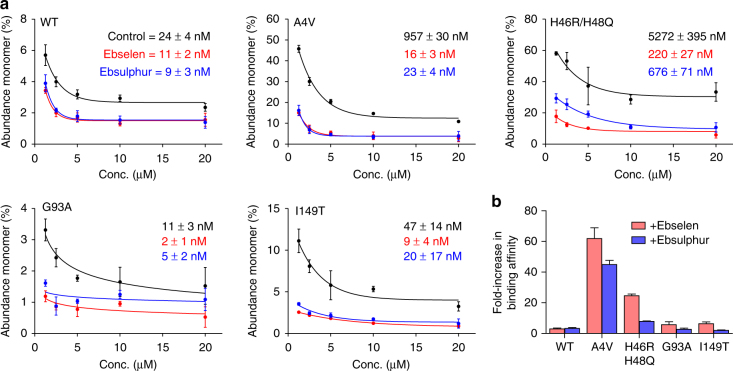
Conjugation of ebselen and ebsulphur to SOD1 variants increases the binding affinity of the dimer. **a** SOD1 variants (wild-type, A4V, H46R/H48Q, G93A and I149T) with ebselen (red), ebsulphur (blue) and without treatment (black) were buffer exchanged into 200 mM NH_4_OAc and subjected to nanoESI mass spectrometry at different protein concentrations under gentle conditions optimized to maintain non-covalent interactions. Curves show the percentage of monomer signal as a function of protein concentration with determined dimer *K*_d_ values inset for each SOD1 variant. Error bars represent the standard deviation of three replicates. **b** The fold-increase in binding affinity of each SOD1 variant when ebselen or ebsulphur is conjugated at Cys111 where error bars represent the upper and lower *K*_d_ values calculated in **a**

Wild-type zinc metalated, disulphide-intact SOD1 was found to have a dimer dissociation constant (*K*_d_) of 24±4 nM and the strength of dimerization is increased slightly by the addition of ebselen and ebsulphur (Fig. 2a). A4V SOD1, which leads to a highly aggressive form of ALS, has a much-weakened dimer interaction resulting in partial monomerization at low micromolar concentrations^29^. This is reflected in an increased dimer *K*_d_, 957±30 nM. However, ebselen and ebsulphur completely restore dimer formation affinity to a wild-type level, *K*_d_ 16±3 and 23±4 nM, respectively (Fig. 2a). A strengthening of the dimer interaction is also observed for ALS mutants G93A and I149T (Fig. 2a). The H46R/H48Q SOD1 artificial copper site mutant displays caricature disease-related characteristics including a very strong propensity to exist in the disulphide-reduced state and weakened affinity for zinc^31^ that leads to complete monomerization at micromolar concentrations (Supplementary Fig. 6). Even in this extreme case, ebselen increases the strength of the dimer, *K*_d_, 5.3±0.4 µM to 220±27 nM, with ebsulphur having a similar but less pronounced effect (Fig. 2a). Overall, all SOD1 mutants showed increased dimer formation affinity, i.e. stabilization, as monomerization is not favoured, when bound to ebselen than ebsulphur, and A4V SOD1 benefitted most with a 60-fold increase in dimer affinity (Fig. 2b).

**Fig. 3:**
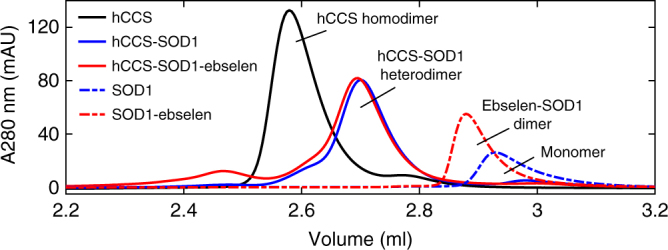
Ebselen does not prevent heterodimer formation with hCCS. Size exclusion chromatograms showing how ebselen binding to C57/146A SOD1 promotes dimerization (blue dashed line to red dashed line) but does not inhibit heterodimeric complex formation with wild-type hCCS, which exists in homodimeric state prior to complexation

The copper chaperone for SOD1, hCCS, is an important part of the cellular machinery that prepares SOD1 for its role in oxidant defence. Modifications to Cys111 that promote SOD1 thermal stability have previously been shown to prevent heterodimer formation between hCCS and SOD1, thus preventing hCCS-catalyzed SOD1 copper acquisition and disulphide formation^20^. In contrast to BMOE and cisplatin, and despite increasing the dimer affinity of SOD1, ebselen does not hinder complex formation between SOD1 and hCCS; the heterodimer readily forms under normal conditions (Fig. 3).

### Ebselen promotes formation of the SOD1 disulphide bond

**Fig. 4:**
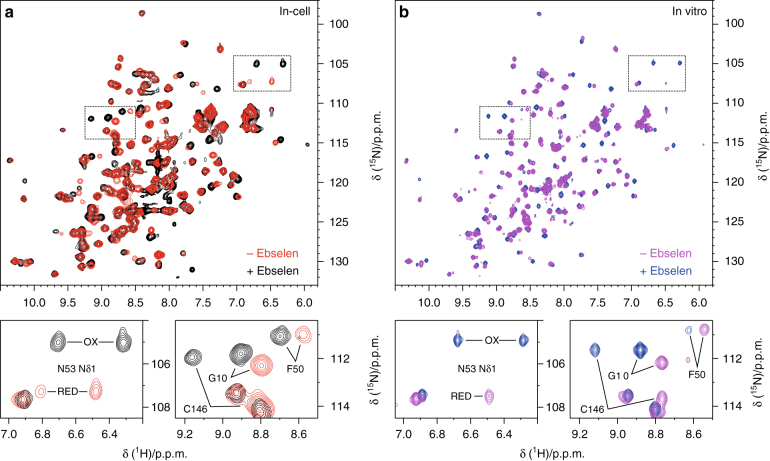
Ebselen efficiently oxidizes SOD1 in living cells. **a** Overlay of ^1^H-^15^N NMR spectra acquired on human cells expressing [U-^15^N] labelled wild-type E,Zn-SOD1 in the absence (red) and in the presence (black) of ebselen in the external medium. **b** Overlay of ^1^H-^15^N NMR spectra acquired on purified, disulphide-reduced [U-^15^N] labelled wild-type E,Zn-SOD1 before (blue) and after (magenta) treatment with 1 equivalent of ebselen. The positions of the enlarged areas (bottom panels) in the whole spectra (top panels) are indicated as dotted lines. Signals affected by the formation of the C57–C146 disulphide bond are labelled in the bottom panels

To determine whether ebselen exerts a protective role against SOD1 in the cytosol, its effect on intracellular SOD1 maturation was investigated in live human cells by in-cell NMR^32,33^. The pathway leading to SOD1 maturation has been recently recapitulated through in-cell NMR studies^34,35^. Beginning with monomeric, disulphide-reduced apo-SOD1, zinc binding occurs spontaneously leading to the formation of reduced E,Zn-SOD1. Copper binding and disulphide bond formation occur subsequently, upon interaction with hCCS^35,36^. Disulphide-reduced E,Zn-SOD1 becomes a stable intermediate species in the cytosol if hCCS is not present in sufficient amount^37^. Ebselen does not change the abundance of wild-type SOD1 and can be applied in the external media to HEK293T cell culture at up to 200 μM without effecting the cell viability (Supplementary Fig. 7). In human cells treated with ebselen, intracellular E,Zn-SOD1 was fully oxidized, showing that ebselen efficiently promotes the formation of the SOD1 intramolecular disulphide bond (between Cys57 and Cys146). In contrast, >95% of the total E,Zn-SOD1 is found in the disulphide-reduced state in untreated cells (Fig. 4a). This effect could be reproduced in vitro by treating reduced E,Zn-SOD1 with one equivalent per monomer of ebselen in an anaerobic environment (Fig. 4b), indicating that a redox reaction occurs directly between ebselen and the reduced Cys57 and Cys146 of SOD1. This mechanism is consistent with the known thiol oxidase activity of ebselen^38^ and would function through the formation of a transient selenylsulphide bond which is then rearranged by selenyl-thiol exchange to yield the SOD1 disulphide and free ebselen selenol (2-hydroseleno-*N*-phenylbenzamide). Interestingly, the ebselen selenol is a potent reducer of hydrogen peroxide and the ultimate product of this reaction is ebselen^39^. Increased oxidative stress could, therefore, promote ebselen recycling.

Ebselen was not found to stably bind Cys111 of either disulphide-oxidized or -reduced SOD1 within the cytosol (Supplementary Figs. 8a and b) likely due to the reduction of the selenylsulphide bond by glutathione, free cysteine or other cellular thiols. This was verified in vitro; treatment of ebselen–SOD1 with thiols at low concentrations (1 mM glutathione or dithiothreitol) at neutral pH resulted in the complete loss of conjugation (Supplementary Fig. 8c).

### Ebselen stabilizes mutant SOD1 in living cells

**Fig. 5:**
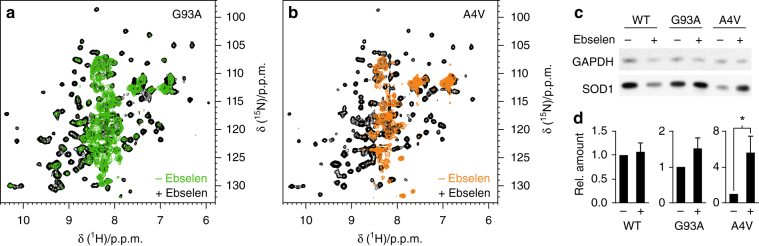
Ebselen stabilizes ALS mutant SOD1 in living cells. **a** Overlay of ^1^H-^15^N NMR spectra acquired on human cells expressing [U-^15^N] labelled SOD1 ALS mutants G93A (**a**) and A4V (**b**) in the absence (green/orange) and in the presence (black) of ebselen in the external medium. NMR signals clustered in the region around 8.3 p.p.m. (^1^H) are characteristic of unstructured SOD1 species. **c** Western blot analysis of cell lysates expressing wild-type and mutant SOD1 in the absence and in the presence of ebselen. Original image Supplementary Fig. 11. **d** Expression levels of wild-type and mutant SOD1 in cell samples treated with ebselen relative to the corresponding untreated samples; error bars represent s.d. (*n* = 3); * indicates significant differences (ratio *t*-test, *p* < 0.05)

**Fig. 6:**
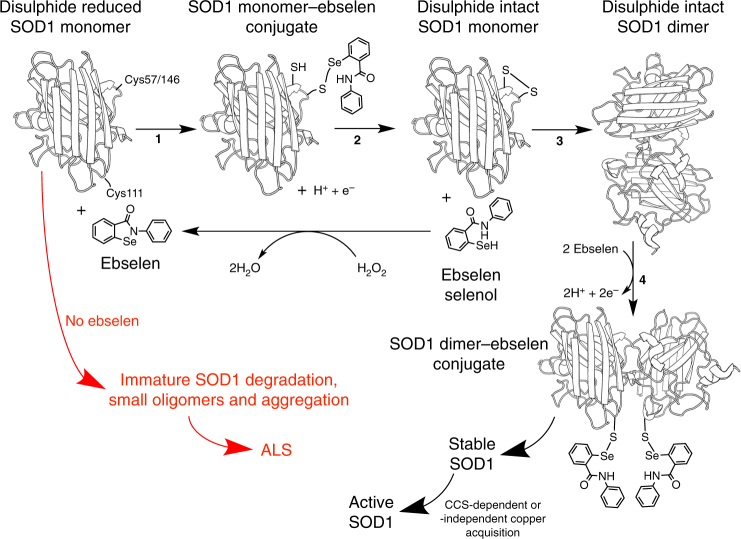
Mechanism of SOD1 stabilization by ebselen. In the absence of ebselen, monomeric, disulphide-reduced, metal-free mutant SOD1 is highly likely to remain globally unfolded and be degraded or accumulate as small toxic oligomers or aggregates, which are characteristic of ALS neural tissues (red). However, in reaction 1, ebselen forms a selenylsulphide bond with either Cys57 or Cys146 on newly translated SOD1. 2. Selenol–disulphide exchange forms the SOD1 intra-subunit disulphide bond and frees the ebselen selenol. Reduction of hydrogen peroxide by ebselen selenol reforms the ebselen heterocycle. This recycling process indicates that relatively small amounts of ebselen could promote SOD1 maturation. 3. SOD1 disulphide formation promotes correct folding, zinc binding and dimerization. 4. Further modification of Cys111 by ebselen prevents monomerization by ALS mutations, thus maintaining stable, active, fully metalated, homodimeric SOD1

Several ALS-linked SOD1 mutants fail to bind zinc in the human cytosol, and accumulate as unstructured species that may act as precursors in the pathogenic aggregation of SOD1^8,9^. Given that the intramolecular disulphide bond is known to increase the structural stability of wild-type and mutant SOD1, we reasoned that the oxidizing effect of ebselen could stabilize intracellular SOD1 mutants. Indeed, treatment with ebselen caused the oxidation of ALS-linked SOD1 mutants G93A and A4V in human living cells, and efficiently stabilized both proteins. This striking effect is evident when comparing the NMR spectra of treated and untreated cells (Fig. 5a, b). In cells treated with ebselen, disulphide intact E,Zn-SOD1 is detected at levels comparable to those of wild-type SOD1, whereas in untreated cells the SOD1 mutants are either largely accumulated as unfolded species (G93A, see Fig. 5a) or barely detected (A4V, possibly due to degradation, see Fig. 5b). This stabilizing effect was further confirmed by western blot analysis of cell lysates, where the expression level of SOD1 mutants was markedly increased upon treatment with ebselen (Fig. 5c). The effect of ebselen treatment on SOD1 aggregates could not be investigated, as the formation of SOD1 aggregates is negligible under the experimental conditions used in this study (Supplementary Fig. 9). Overall, these data indicate that the action of ebselen in stabilizing intracellular SOD1 occurs through a selenol–thiol exchange mechanism similar to that deployed by hCCS. The two modes of action targeting disulphide-intact and disulphide-reduced SOD1 (Fig. 6) are, however, not mutually exclusive and both could be exploited in future drug developments against ALS.

## Discussion

ALS-related SOD1 mutations are distributed throughout the molecule’s structure with two exceptions: the disulphide sub-loop and the β-strand 3. The stability of the dimeric structure is a key factor that determines the pathogenicity of the ALS SOD1 mutants with the interface mutants A4V and I113T causing some of the most aggressive forms of the disease. Dimerization, disulphide bond formation and metal ion acquisition are the canonical SOD1 PTM steps en route to forming a stable and active enzyme^5–7^. Each PTM contributes to both stability and activity. This appears to extend to the bronchial inflammation disease asthma, where the wild-type SOD1 disulphide is reduced as a result of an imbalanced oxidized/reduced glutathione ratio. Ultimately, disulphide-reduced, metal-free SOD1 accumulates in amorphous and fibrillar aggregates in the cytoplasm and mitochondria of neurons and astroglia of ALS patients and mutant SOD1 transgenic mice^10,11,40^. The affinity of mutant SOD1 for metals is inherently difficult to directly influence. Folding, disulphide bonding and dimerization are, however, open to therapeutic manipulation. Here we have shown that ebselen has astonishing capacities for correcting several aspects of aberrant mutant SOD1 maturation with implications for the treatment of ALS and asthma.

We have quantified the dimer dissociation constants of wild-type SOD1, and several disease-related mutants, in non-denaturing conditions using native MS. While A4V and the artificial copper site mutant H46R/H48Q have a 40-fold and 220-fold reduction in their ability to form a dimer, respectively, G93A and I149T have wild-type-like dimer interactions. This highlights the likelihood that SOD1 monomerization acts in concert with reduced copper or zinc affinity, thermal instability, disulphide reduction and local or global unfolding as the toxic cue. Using a combination of protein crystallography and native MS, we have determined both the binding site and pose of two novel ligands, directly quantifying their effect on SOD1 dimerization and predicting their mode of action. We conclude that ebselen binding to Cys111 restores the dimer formation affinity of A4V SOD1 to that of the wild-type. The effect is mediated by π–π stacking between ebselen or ebsulphur aromatic rings and increases the dimer binding energy by exclusion of water. SOD1 dimer formation affinity is increased by both ebselen and ebsulphur binding irrespective of the SOD1 variant involved; π–π stacking is often used as a mechanism by which to structurally stabilize and dictate the structure of proteins, nucleic acids and their complexes. However, we are not aware of a system in which ligand binding extends an interface surface, thereby increasing the strength of oligomerization. Aromatic stacking is observed in the consecutive nitrogenous bases of DNA. This effect provides roughly 1 kcal/mol of free energy per base and encourages adoption of a helical structure but allows strand separation and unwinding during replication and transcription. We believe a similar, relatively small contribution is exerted by ebselen and ebsulphur at the SOD1 dimer interface, thereby promoting homodimerization but also allowing heterodimerization when presented with hCCS.

As is the case for cisplatin and bis-maleimide derivatives, ebselen and ebsulphur are promiscuous thiol oxidases and form conjugates with a variety of proteins. However, the reaction with the target cysteine is reversible and likely to be stripped by cellular reductants such as glutathione. Indeed, our in-cell and in vitro NMR observations show that ebselen is not stably bound to Cys111 in the reducing environment of the cytosol. Nevertheless, such reversible conjugation constitutes a novel means to modulate the behaviour of SOD1 associated with ALS, and may become useful if the target is in less thiol-reducing environments at the point of intervention, such as the mitochondrial inter-membrane space or the extracellular matrix. In the former, fine-tuning of glutaredoxin activity^41^ allows for a high frequency of disulphide bonding to occur and several instances of selenylsulphide bonding^42^. In the latter, the lower glutathione concentration (micromolar rather than millimolar) and oxidizing redox potential^43^ makes disulphide bonding more favourable and would encourage the formation of the Cys111–ebselen or ebsulphur conjugate. SOD1 mitochondrial pathology^25^ and extracellular transmission of misfolded SOD1^44^ are important aspects of ALS pathogenesis and spread of the disease throughout the motor system. Inhibiting these effects by stabilization of SOD1 is therefore a good candidate for slowing the disease course and has been shown effective for ebselen and other small molecules^25,45^. Moreover, ebselen and ebsulphur are more amenable scaffolds from which to grow into the SOD1 dimer groove and engineer specificity than cisplatin or BMOE, whose mode of action is unclear or require covalent dimer tethering respectively.

In recent years, the toxicity of large, insoluble SOD1 aggregates has been questioned. Monomers which are unable to bind zinc, fold and dimerize or small oligomers with non-native interface regions are now thought to be the origin of SOD1 toxicity. In most cases, these inherently unstable species are cleared by the cellular degradation machinery leading to a reduction in the abundance and activity of mutant SOD1. Reduction in the amount of erythrocyte SOD1 is in fact strongly associated with ALS mutations^46–48^. However, a fraction of the mutant SOD1 does not undergo controlled degradation. Instead, it sequesters and inhibits the proteasome and chaperone-mediated folding pathways. Ultimately these species go on to form large insoluble aggregates but the damage has already been done and cell death ensues. While ebselen does not appear to form a stable covalent bond with SOD1 Cys111 in the reducing environment of the cytosol, it does very efficiently redirect the fate of these small toxic species. We have shown, based on in-cell NMR characterization, that ebselen restores the mutant SOD1 PTM pathway by promoting the formation of the intra-subunit disulphide bond. The mode of action of ebselen in cells has three possibilities: (1) An indirect effect on intracellular redox which promotes disulphide formation. (2) Increased activity of the SOD1 disulphide conferring chaperone protein hCCS. (3) Direct redox reaction between ebselen and SOD1 cysteines involved in the intra-subunit disulphide bond. While the first two hypotheses cannot be ruled out, we have shown the latter to occur spontaneously in vitro, in the absence of additional thiol-containing molecules. Therefore, ebselen likely reacts with one of the free Cys57 or Cys146 sulphydryls on SOD1, forming a transient selenyl-sulphide which is then easily rearranged by selenyl-thiol exchange to form the intra-subunit disulphide bond with the release of ebselen selenol. Disulphide bond formation greatly enhances the folding stability of mutant SOD1, an effect also observed for prokaryotic Cu/ZnSOD^49^, and improves zinc binding affinity. Thus, the level of soluble mutant protein, which is constituted by SOD1 not swiftly degraded or aggregated, is increased.

In summary, by strongly promoting SOD1 disulphide formation, ebselen facilitates correct folding and zinc acquisition of mutant SOD1 in the cytosol, while in less reducing environments it greatly enhances the SOD1 dimer affinity (Fig. 6). Ebselen can, therefore, be considered a true pharmacological chaperone for SOD1. These positive effects are important milestones in SOD1-ALS drug development. In addition, Ebselen is known to inhibit inducible nitric oxide synthase, detoxify peroxynitrite and combat glutamate-induced excitotoxicity^50–52^. This antioxidant behaviour, together with the pharmacological similarities with edaravone, indicate ebselen is likely to have positive secondary pharmacology as well as the primary pharmacological chaperone role investigated here. In addition, ebselen is well tolerated in humans and can be formulated for oral administration. Several other drug-like molecules have been shown to negatively affect the interaction of SOD1 with hCCS^20^. For SOD1 to perform its function it must be copper metalated and disulphide oxidized; a function largely performed by hCCS which takes E,Zn-SOD1^S−H^ as a substrate and yields active and highly stable SOD1. While cisplatin and BMOE inhibit or completely prevent the interaction with hCCS, respectively, ebselen-bound SOD1 is free to heterodimerize with its protein chaperone in vitro despite the increased dimer affinity engendered by ebselen binding. If the reversibility of ebselen binding can be fine-tuned through rational structurally informed design, the implication is that SOD1’s toxic characteristics could be modulated in vivo over long time-courses for the treatment of ALS and asthma without damaging our response to oxidative stress.

## Methods

### Expression and purification of SOD1

Mutant SOD1-pET303C expression plasmids were generated by site-directed mutagenesis (SDM) using oligonucleotides with sequences found in Supplementary Table 1. Recombinant wild-type and mutant SOD1 were expressed in BL21 (DE3) *Escherichia coli* in LB media for 16 h at 25 and 18 °C, respectively, followed by purification on diethylaminoethyl cellulose^17^. Ebselen-bound SOD1 was produced by adding the compound from a DMSO stock. Excess compound was removed by dialysis. H46R/H48Q SOD1 was analyzed by size exclusion chromotography (SEC) as described previously^20^. Disulphide-reduced E,Zn-SOD1 was obtained by adding 50 mM DTT to apo-SOD1 in phosphate-buffered saline (PBS) and incubating for 40 min at 37 °C. DTT was subsequently removed under anaerobic conditions and 1 equivalent of zinc was added.

### Crystallization and structure solution

Crystals of as-isolated Zn-bound SOD1 were produced using previously identified conditions with the protein pre-conjugated with the ligand. Crystals were frozen in 3.2 M (NH_4_)_2_SO_4_ as a cryoprotectant and data were collected on I02 at Diamond Light Source and Proxima1 at SOLEIL for ebsulphur and ebselen-bound SOD1, respectively. Diffraction images were indexed and integrated using iMosflm^53^ and data were scaled using Aimless in the CCP4 suite^54^. Rigid body refinement was performed using unmodified wild-type SOD1 from the same crystal unit cell (PDB: 2V0A). Models were manually refined using Coot^55^ followed by cycles of restrained refinement in Refmac5^56^. Restraints for both ebselen and ebsulphur were produced using JLigand^57^. Maps were produced with Phenix using simulated annealing^58^. Crystallographic statistics are shown in Table 1. The structures of ebselen- and ebsulphur-bound SOD1 are deposited in the PDB with accession numbers 5O40 (10.2210/pdb5O40/pdb) and 5O3Y (10.2210/pdb5O3Y/pdb).

**Table 1:** Crystallographic statistics for SOD1-bound ebselen and ebsulphur structures

	wtSOD1-ebselen	wtSOD1-ebsulphur
*Data collection*		
Space group	*P* 2_1_	*P* 2_1_
Cell dimensions		
*a*, *b*, *c* (Å)	38.10, 67.84, 51.04	38.05, 67.96, 51.18
*α*, *β*, *γ* (°)	90.00, 106.35, 90.00	90.00, 106.73, 90.00
Resolution (Å)	49.01–1.50 (1.52–1.50)^a^	67.84–1.30 (1.33–1.30)^a^
*R*_merge_	11.7 (59.4)	6.4 (56.6)
*I*/ *σI*	5.9 (1.5)	7.1 (1.4)
CC_1/2_ (%)	0.991 (0.494)	0.991 (0.429)
Completeness (%)	99.9 (99.5)	98.8 (97.7)
Redundancy	3.1 (3.1)	3 (2.8)
*Refinement*		
No. reflections	39,891	60,537
*R*_work_/*R*_free_	16.05/21.24	14.53/18.10
No. atoms		
Protein	2241	2274
Ligand/ion	46	46
Water	309	311
*B*-factors		
Protein	12.35	15.45
Ligand/ion	10.32	15.47
Water	29.27	35.38
R.M.S. deviations		
Bond lengths (Å)	0.013	0.015
Bond angles (°)	1.607	1.683
PDB code	5O40	5O3Y

^a^Values in parentheses are for the highest-resolution shell

### Native MS

Purified SOD1 samples with pre-conjugated ligand were buffer exchanged to remove free ligand and diluted to 5 µM in 100 mM NH_4_OAc, 50% acetonitrile and 2.5% formic acid. Samples were incubated for 30 min at room temperature before being nano-electrosprayed. Spectra were externally calibrated using a solution of caesium iodide (10 mg/mL) in 50% *n*-propanol and masses were determined using Masslynx 4.1 software (Waters, UK).

Native MS analysis was performed using a SYNAPT G1 HDMS (Waters, UK) with parameters set according to previous work^30^. Briefly, purified recombinant SOD1 protein was buffer exchanged into 200 mM NH_4_OAc (pH 6.8) using centrifugal concentrators with a 10-kDa molecular weight cut-off. Buffer exchanged SOD1 was diluted to working concentrations in NH_4_OAc, loaded into gold-coated borosilicate capillaries (made in-house), and subjected to nano-electrospray ionization (*n* = 1 electrosprays for 3 separate dilutions). All spectra were externally calibrated using 1 mg/mL caesium-iodide in 50% *n*-propanol (Supplementary Fig. 10), and were processed using Masslynx 4.1 software.

### Dissociation constant determination

Dissociation constants were determined according to Rose et al^59^. All spectra were smoothed and processed using the same parameters. Dimer dissociation can be simply defined as a dimer (*M*_2_) dissociating into monomers (*M*) with conservation of mass, meaning that a single dimer dissociates into two monomers:

$$M_2 \leftrightarrow 2M.$$

Despite MS not being quantitative, the detected intensity of each species comprising a complex in equilibrium is dependent upon their relative concentrations in solution. Therefore, by measuring the intensity of monomers and dimers across a range of known protein concentrations, it is possible to determine the proportion of monomers and dimers present in solution at a given concentration.

Briefly, the intensity, as determined by the area under the peak, of all peaks relating to SOD1 monomers (*I*_M_) and dimers (*I*_D_) were summed to give total signal intensity (*I*_T_):

1 $$I_{\rm M} + I_{\rm D} = I_{\rm T}.$$

The proportion of monomer signal (*P*_M_) was determined by dividing the intensity of the monomeric species (*I*_M_) by the total signal intensity (*I*_T_):

2 $$\frac{{I_{\rm M}}}{{I_{\rm T}}} = P_{\rm M}.$$

The concentration of monomer at equilibrium ([*M*]_eq_) was determined by multiplying the proportion of monomer signal (*P*_M_) by the total protein concentration in micromolar ([*P*_0_]):

3 $$P_{\rm M} \times \left[ {P_{\rm 0}} \right] = [M]_{{\rm eq}}.$$

The concentration of dimer at equilibrium ([*D*]_eq_) is defined as

4 $$\frac{{\left( {\left[ {P_{\rm 0}} \right] - \left[ M \right]_{{\rm eq}}} \right)}}{2} = [D]_{{\rm eq}}.$$

*K*_d_ values were derived from the slope of a linear regression fit of the plot of [*D*]_eq_ against [*M*]^2^_eq_ (fitting performed with Prism 5.0, GraphPad Software, Inc.) (Supplementary Fig. 5). Data were only accepted if the line of best fit gave least-squares regression, *R*^2^ < 0.85.

### Size-exclusion chromatography

We used the artificial disulphide knockout SOD1 mutant C57A/C146A to assay heterodimer formation with hCCS. This allowed us to bind ebselen to Cys111 only while maintaining the conditions necessary for complexation; 25 μM SOD1 was incubated with 100 μM ebselen dissolved in 100% DMSO for 3 h at 20 ^o^C. hCCS was desalted into ligand-free, nitrogen-purged buffer. The complex was formed with 250 pmol of both SOD1 and hCCS and was observed as described previously^20^. Ebselen-free SOD1 was also treated with DMSO.

### Human cell culture and transfection

HEK293T (ATCC CRL-3216) cells were maintained in DMEM high glucose (Life Technologies) supplemented with L-glutamine, antibioticss (penicillin and streptomycin) and 10% FBS (Gibco) in uncoated 75 cm^2^ plastic flasks, and were grown at 37 °C, 5% CO_2_ in a humidified atmosphere. Cells were transiently transfected with SOD1 cDNA (either wild-type or mutant) cloned in the pHLsec plasmid^60^ using polyethylenimine (PEI)^4^ with a DNA:PEI ratio of 1:1 (50 μg/flask DNA, 50 μg/flask PEI). For wild-type SOD1, 25 μg/flask of cDNA was used (mixed with an equal amount of empty DNA vector) to compensate for the lower expression level of the mutants. [U-^15^N]-BioExpress6000 medium (Cambridge Isotope Laboratories) was used for in-cell NMR samples, supplemented with 2% FBS, antibiotics and 10 µM of ZnSO_4_.

### Treatment with ebselen

The cellular response to ebselen in terms of cell viability and SOD1 expression was assessed by trypan blue staining and western blot of HEK293T cells overexpressing wild-type SOD1 and subsequently treated for 24 h with increasing concentrations of ebselen (Supplementary Fig. 7). While SOD1 expression levels were not affected (Supplementary Fig. 7a), cell viability was markedly decreased above 200 µM ebselen (Supplementary Fig. 7b). Therefore, ebselen treatment was performed by adding 200 µM ebselen to the external medium 24 h before collecting the cells.

### In-cell NMR sample preparation

Samples for in-cell NMR were prepared following a reported protocol^61^. Briefly, transfected cells were detached with trypsin, suspended in DMEM + 10% FBS, washed once with PBS and re-suspended in one pellet volume of DMEM supplemented with 90 mM glucose, 70 mM HEPES and 20% D_2_O. The cell suspension was transferred in a 3-mm Shigemi NMR tube, which was gently spun to sediment the cells. Cell viability before and after NMR experiments was assessed by trypan blue staining. After the NMR experiments, the cells were collected and the supernatant was checked for protein leakage by NMR.

### NMR experiments

NMR spectra, both in-cell and in vitro, were collected at 308 K with a 950-MHz Bruker Avance III spectrometer, equipped with a CP TCI CryoProbe. For cell samples, 2D ^1^H-^15^N SOFAST HMQC^62^ spectra (~1 h) were recorded with 64 scans, 2048 points, 128 increments and a 0.3-s recycle delay. For in vitro NMR, samples of 130 µM E,Zn-SOD1 in PBS, pH 7.4, were analyzed in 3 mm NMR tubes. 2D ^1^H-^15^N SOFAST HMQC spectra (~40 m) were recorded with 32 scans, 2048 points, 192 increments and a 0.3-s recycle delay.

The NMR spectra collected were processed with Topspin NMR data-processing software. The in-cell NMR spectra were further processed by subtracting a spectrum of cells transfected with empty vector, acquired in the same experimental conditions, to eliminate the signals arising from partial ^15^N labelling of other cellular components.

### Western blot analysis

Cell lysates were prepared by freeze–thaw cycles in PBS buffer followed by centrifugation to remove the insoluble fraction. SOD1 (both wild-type and mutants) was stained with a rabbit polyclonal anti-SOD1 antibody (Abcam: ab16831, diluted 1:2000 to 0.5 mg/mL); a goat anti-rabbit IgG (whole molecule)-peroxidase secondary antibody (Sigma:A0545) was used, diluted at 1:80,000. SOD1 expression levels were normalized to GAPDH, which was stained with a rabbit polyclonal anti-GAPDH antibody (Abcam: ab9485, diluted at 1:2000), or to beta-tubulin, which was stained with a rabbit polyclonal anti-beta Tubulin antibody (Abcam: ab6046, diluted at 1:500). LiteAblot EXTEND chemiluminescent substrate (EuroClone) was used for detection.

### SOD1 aggregation assay

SOD1 aggregation assay was performed following a reported protocol^63^. Briefly, cells were lysed by freeze–thaw cycles in PBS buffer followed by centrifugation. The supernatant was collected as the soluble fraction, while the pellet (insoluble fraction) was further processed to extract the detergent-soluble fraction. The pellet was washed thrice in PBS buffer, resuspended in 10 mM Tris-HCl pH 8.0, 1 mM EDTA pH 8.0, 100 mM NaCl, 0.5% Nonidet P-40, and centrifuged at 16,000×*g* for 30 min. The supernatant was collected as detergent-soluble fraction.

### Data availability

Crystal structures are deposited in the Protein Data Bank under accession numbers 5O40 and 5O3Y. Other relevant data are available from the corresponding authors upon reasonable request.

## Electronic supplementary material

Supplementary Information

## References

[1] Rosen DR (1993). Mutations in Cu/Zn superoxide dismutase gene are associated with familial amyotrophic lateral sclerosis. Nature.

[2] Kerman A (2010). Amyotrophic lateral sclerosis is a non-amyloid disease in which extensive misfolding of SOD1 is unique to the familial form. Acta Neuropathol..

[3] Yerbury JJ (2016). Walking the tightrope: proteostasis and neurodegenerative disease. J. Neurochem..

[4] McCord JM, Fridovich I (1969). Superoxide dismutate an enzymic function for erthrocuprein (Hemocurprein). J. Biol. Chem..

[5] Carrico RJ, Deutsch HF (1970). The presence of zinc in human cytocuprein and some properties of the apoprotein. J. Biol. Chem..

[6] Wood E, Dalgleish D, Bannister W (1971). Bovine erythrocyte cupro-zinc protein. Eur. J. Biochem..

[7] Forman HJ, Fridovich I (1973). On the stability of bovine superoxide dismutase: thef effects of metals. J. Biol. Chem..

[8] Lang L (2015). SOD1 aggregation in ALS mice shows simplistic test tube behavior. Proc. Natl Acad. Sci. USA.

[9] Luchinat E (2014). In-cell NMR reveals potential precursor of toxic species from SOD1 fALS mutants. Nat. Commun..

[10] Jonsson PA (2006). Disulphide-reduced superoxide dismutase-1 in CNS of transgenic amyotrophic lateral sclerosis models. Brain.

[11] Bourassa MW, Brown HH, Borchelt DR, Vogt S, Miller LM (2014). Metal-deficient aggregates and diminished copper found in cells expressing SOD1 mutations that cause ALS. Front. Aging Neurosci..

[12] De Raeve H (1997). Decreased Cu,Zn-SOD activity in asthmatic airway epithelium: correction by inhaled corticosteroid in vivo. Am. J. Physiol..

[13] Ghosh S (2013). Disulfide bond as a switch for copper-zinc superoxide dismutase activity in asthma. Antioxid. Redox Signal..

[14] Wang Q, Johnson JL, Agar NYR, Agar JN (2008). Protein aggregation and protein instability govern familial amyotrophic lateral sclerosis patient survival. PLoS Biol..

[15] Pratt AJ (2014). Aggregation propensities of superoxide dismutase G93 hotspot mutants mirror ALS clinical phenotypes. Proc. Natl Acad. Sci. USA.

[16] McAlary L, Aquilina JA, Yerbury JJ (2016). Susceptibility of mutant SOD1 to form a destabilized monomer predicts cellular aggregation and toxicity but not in vitro aggregation propensity. Front. Neurosci..

[17] Wright GSA, Antonyuk SV, Kershaw NM, Strange RW, Hasnain SS (2013). Ligand binding and aggregation of pathogenic SOD1. Nat. Commun..

[18] Auclair JR, Boggio KJ, Petsko GA, Ringe D, Agar JN (2010). Strategies for stabilizing superoxide dismutase (SOD1), the protein destabilized in the most common form of familial amyotrophic lateral sclerosis. Proc. Natl Acad. Sci. USA.

[19] Banci L (2012). Interaction of cisplatin with human superoxide dismutase. J. Am. Chem. Soc..

[20] Wright GSA, Antonyuk SV, Hasnain SS (2016). A faulty interaction between SOD1 and hCCS in neurodegenerative disease. Sci. Rep..

[21] Schewe T (1995). Molecular actions of ebselen - an antiinflammatory antioxidant. Gen. Pharmacol..

[22] Yamaguchi T (1998). Ebselen in acute ischemic stroke: a placebo-controlled, double-blind clinical trial. Stroke.

[23] Singh N (2013). A safe lithium mimetic for bipolar disorder. Nat. Commun..

[24] Saito I (1998). Neuroprotective effect of an antioxidant, ebselen, in patients with delayed neurological deficits after aneurysmal subarachnoid hemorrhage. Neurosurgery.

[25] Wood-Allum CA (2006). Impairment of mitochondrial anti-oxidant defence in SOD1-related motor neuron injury and amelioration by ebselen. Brain.

[26] Shim JS, Liu JO (2014). Recent advances in drug repositioning for the discovery of new anticancer drugs. Int. J. Biol. Sci..

[27] Ito H (2008). Treatment with edaravone, initiated at symptom onset, slows motor decline and decreases SOD1 deposition in ALS mice. Exp. Neurol..

[28] Hough MA (2004). Dimer destabilization in superoxide dismutase may result in disease-causing properties: structures of motor neuron disease mutants. Proc. Natl Acad. Sci. USA.

[29] Ray SS (2004). An intersubunit disulfide bond prevents in vitro aggregation of a superoxide dismutase-1 mutant linked to familial amytrophic lateral sclerosis. Biochemistry.

[30] McAlary L, Yerbury JJ, Aquilina JA (2013). Glutathionylation potentiates benign superoxide dismutase 1 variants to the toxic forms associated with amyotrophic lateral sclerosis. Sci. Rep..

[31] Winkler DD (2009). Structural and biophysical properties of the pathogenic SOD1 variant H46R/H48Q. Biochemistry.

[32] Freedberg DI, Selenko P (2014). LiveCell NMR. Annu. Rev. Biophys..

[33] Luchinat E, Banci L (2017). In-cell NMR: a topical review. IUCrJ.

[34] Banci L, Barbieri L, Bertini I, Cantini F, Luchinat E (2011). In-cell NMR in E. coli to monitor maturation steps of hSOD1. PLoS ONE.

[35] Banci L (2013). Atomic-resolution monitoring of protein maturation in live human cells by NMR. Nat. Chem. Biol..

[36] Banci L (2012). Human superoxide dismutase 1 (hSOD1) maturation through interaction with human copper chaperone for SOD1 (hCCS). Proc. Natl Acad. Sci. USA.

[37] Mercatelli E, Barbieri L, Luchinat E, Banci L (2016). Direct structural evidence of protein redox regulation obtained by in-cell NMR. Biochim. Biophys. Acta.

[38] Sakurai T (2006). Ebselen, a seleno-organic antioxidant, as an electrophile. Chem. Res. Toxicol..

[39] Zhao R, Holmgren A (2002). A novel antioxidant mechanism of ebselen involving ebselen diselenide, a substrate of mammalian thioredoxin and thioredoxin reductase. J. Biol. Chem..

[40] Karch CM, Prudencio M, Winkler DD, Hart PJ, Borchelt DR (2009). Role of mutant SOD1 disulfide oxidation and aggregation in the pathogenesis of familial ALS. Proc. Natl Acad. Sci. USA.

[41] Kojer K, Peleh V, Calabrese G, Herrmann JM, Riemer J (2015). Kinetic control by limiting glutaredoxin amounts enables thiol oxidation in the reducing mitochondrial intermembrane space. Mol. Biol. Cell..

[42] Kim A (2014). A panoramic overview of mitochondria and mitochondrial redoxbiology. Toxicol. Res..

[43] Jones DP (2000). Redox state of glutathione in human plasma. Free Radic. Biol. Med..

[44] Grad LI (2014). Intercellular propagated misfolding of wild-type Cu/Zn superoxide dismutase occurs via exosome-dependent and -independent mechanisms. Proc. Natl Acad. Sci. USA.

[45] Pokrishevsky E, Hong RH, Mackenzie IR, Cashman NR (2017). Spinal cord homogenates from SOD1 familial amyotrophic lateral sclerosis induce SOD1 aggregation in living cells. PLoS ONE.

[46] Deng HX (1993). Amyotrophic lateral sclerosis and structural defects in Cu, Zn superoxide dismutase. Science.

[47] Borchelt DR (1995). Superoxide dismutase 1 subunits with mutations linked to familial amyotrophic lateral sclerosis do not affect wild type subunit function. J. Biol. Chem..

[48] Sato T (2005). Rapid disease progression correlates with instability of mutant SOD1 in familial ALS. Neurology.

[49] Sakurai Y, Anzai I, Furukawa Y (2014). A primary role for disulfide formation in the productive folding of prokaryotic Cu,Zn-superoxide dismutase. J. Biol. Chem..

[50] Zembowicz A, Hatchett RJ, Radziszewski W, Gryglewski RJ (1993). Inhibition of endothelial nitric oxide synthase by ebselen. prevention by thiols suggests the inactivation by ebselen of a critical thiol essential for the catalytic activity of nitric oxide. J. Pharmacol. Exp. Ther..

[51] Masumoto H, Sies H (1996). The reaction of Ebselen with peroxynitrite. Chem. Res. Toxicol..

[52] Porciúncula LO, Rocha JBT, Boeck CR, Vendite D, Souza DO (2001). Ebselen prevents excitotoxicity provoked by glutamate in rat cerebellar granule neurons. Neurosci. Lett..

[53] Battye TGG, Kontogiannis L, Johnson O, Powell HR, Leslie AGW (2011). iMOSFLM: a new graphical interface for diffraction-image processing with MOSFLM. Acta Crystallogr. D Biol. Crystallogr..

[54] C.C.P. Number 4. The CCP4 suite: programs for protein crystallography. *Acta Crystallogr. D Biol. Crystallogr*. **D50**, 760–763 (1994). 10.1107/S0907444994003112 15299374

[55] Emsley P, Lohkamp B, Scott WG, Cowtan K (2010). Features and development of Coot. Acta Crystallogr. D Biol. Crystallogr..

[56] Murshudov GN (2011). REFMAC5 for the refinement of macromolecular crystal structures. Acta Crystallogr. D Biol. Crystallogr..

[57] Lebedev AA (2012). JLigand: a graphical tool for the CCP4 template-restraint library. Acta Crystallogr. D Biol. Crystallogr..

[58] Pražnikar J, Afonine PV, Gunčar G, Adams PD, Turk D (2009). Averaged kick maps: less noise, more signal and probably less bias. Acta Crystallogr. D Biol. Crystallogr..

[59] Rose RJ (2011). Quantitative analysis of the interaction strength and dynamics of human IgG4 half molecules by native mass spectrometry. Structure.

[60] Aricescu AR, Lu W, Jones EY (2006). A time- and cost-efficient system for high-level protein production in mammalian cells. Acta Crystallogr. D Biol. Crystallogr..

[61] Barbieri L, Luchinat E, Banci L (2016). Characterization of proteins by in-cell NMR spectroscopy in cultured mammalian cells. Nat. Protoc..

[62] Schanda P, Brutscher B (2005). Very fast two-dimensional nmr spectroscopy for real-time investigation of dynamic events in proteins on the time scale of seconds. J. Am. Chem. Soc..

[63] Cozzolino M (2008). Cysteine 111 affects aggregation and cytotoxicity of mutant Cu,Zn-superoxide dismutase associated with familial amyotrophic lateral sclerosis. J. Biol. Chem..

