# Supplementary material for: The cysteine-reactive small molecule ebselen facilitates effective SOD1 maturation
Source: Nat Commun. 2018 Apr 27;9:1693. doi: 10.1038/s41467-018-04114-x (PMC5923229; doi:10.1038/s41467-018-04114-x)
Supplement: Supplementary file 1 — Supplementary Information [file 41467_2018_4114_MOESM1_ESM.pdf]

Supplementary Information

The cysteine-reactive small molecule ebselen facilitates effective SOD1 maturation

Capper *et al*.

Supplementary Information

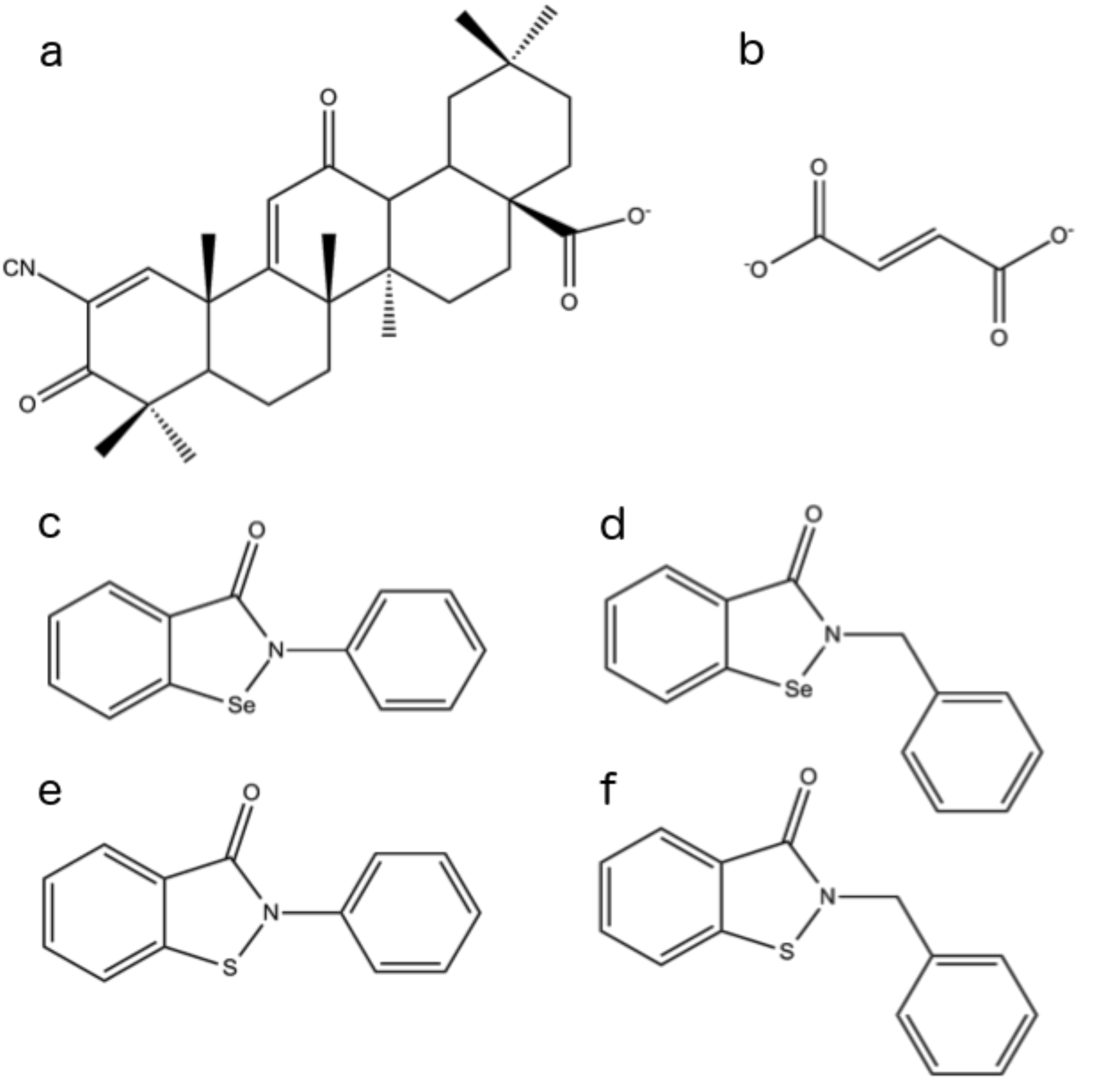

Supplementary Figure 1 | Cysteine reactive compounds: (a) CDDO/Bardoxolone **(b)** fumarate **(c)** ebselen (2-phenylbenzo[d][1,2]selenazol-3(2H)-one) **(d)** 2-benzylbenzo[d][1,2]selenazol-3(2H)-one **(e)** ebsulphur (2-phenylbenzo[d]isothiazol-3(2H)-one) **(f)** 2-benzylbenzo[d]isothiazol-3(2H)-one.

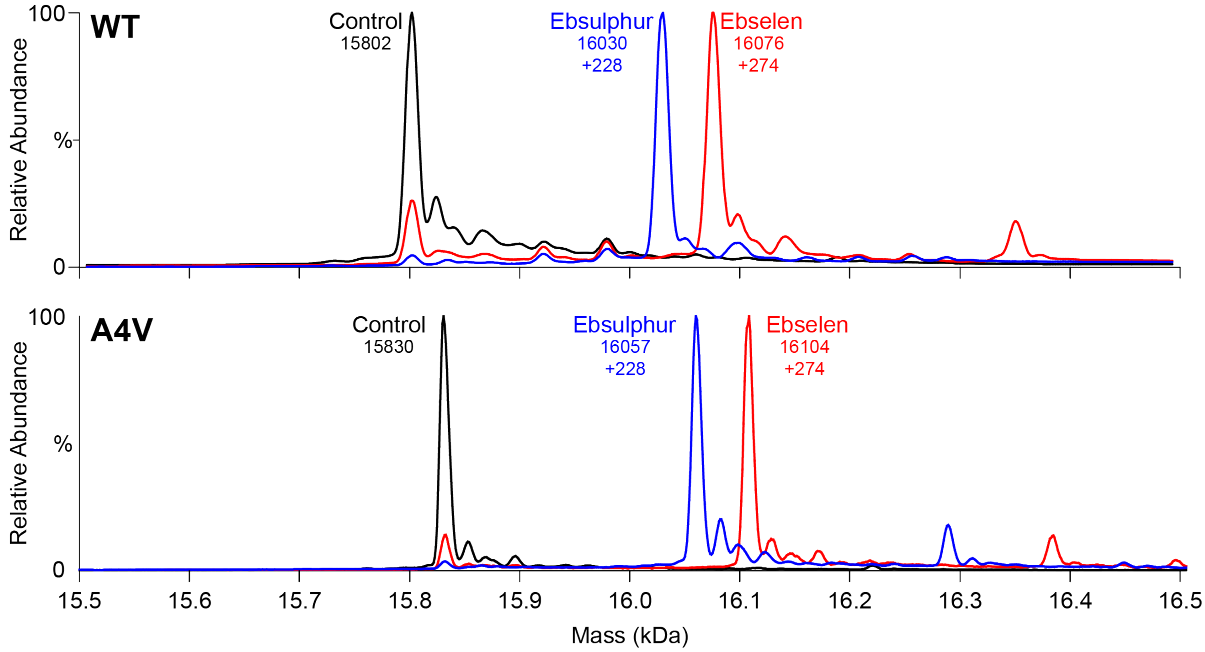

Supplementary Figure 2 | Covalent binding of ebselen and ebsulphur to SOD1 measured by mass spectrometry. Mass transforms from acid denatured wild-type and A4V SOD1 with no ligand (black), ebselen (red), and ebsulphur (blue) with measured masses (in daltons) with the difference in mass compared to no-ligand control. The most abundant species in the ebselen and ebsulphur samples was covalently ligand-modified SOD1.

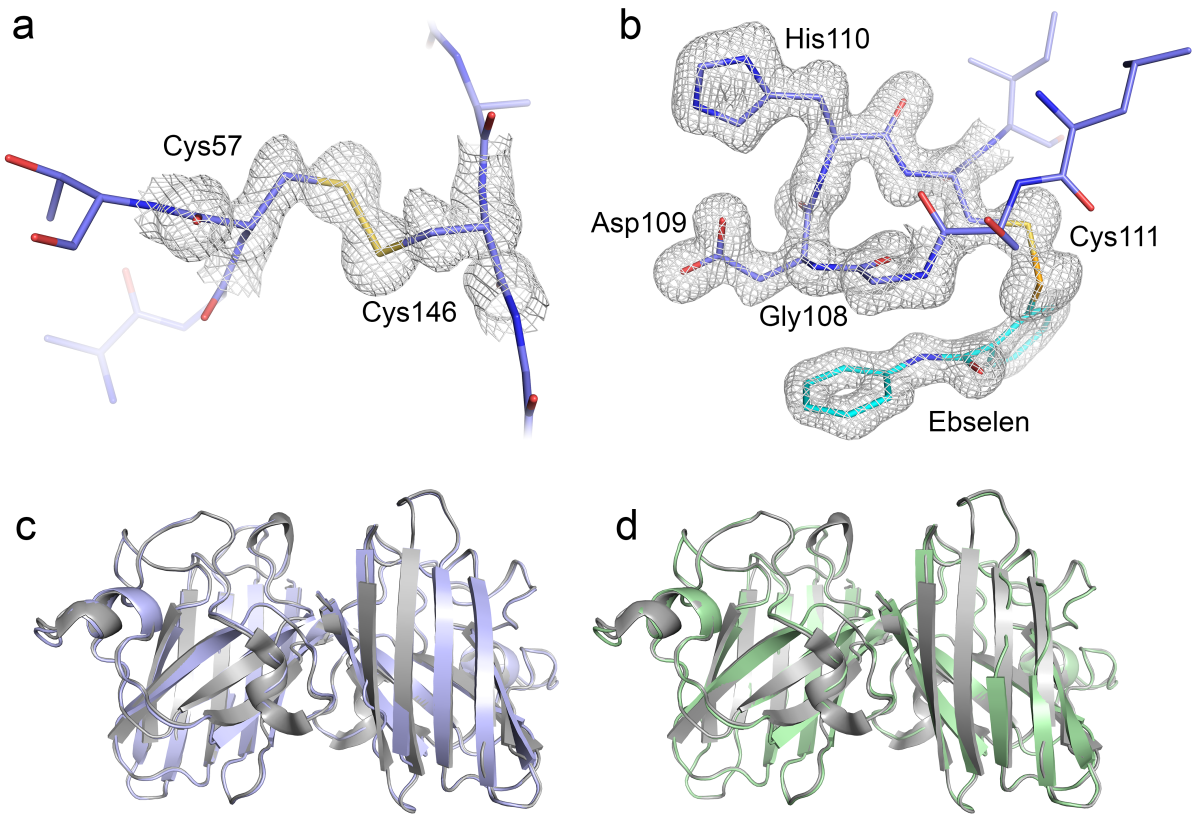

Supplementary Figure 3 | Ligand binding does not change the structure of SOD1. 2Fo-Fc electron density maps contoured at 1 σ showing (**a**) the intact disulphide bond between Cys57 and Cys146 and (**b**) positioning of ebselen close to the SOD1 loop VI. Comparison of wild-type SOD1 (PDB 2C9V) bound to (**c**) ebselen and (**d**) ebsulphur respectively show very little difference in overall three-dimensional structure and have an overall RMSD of 0.24 Å.

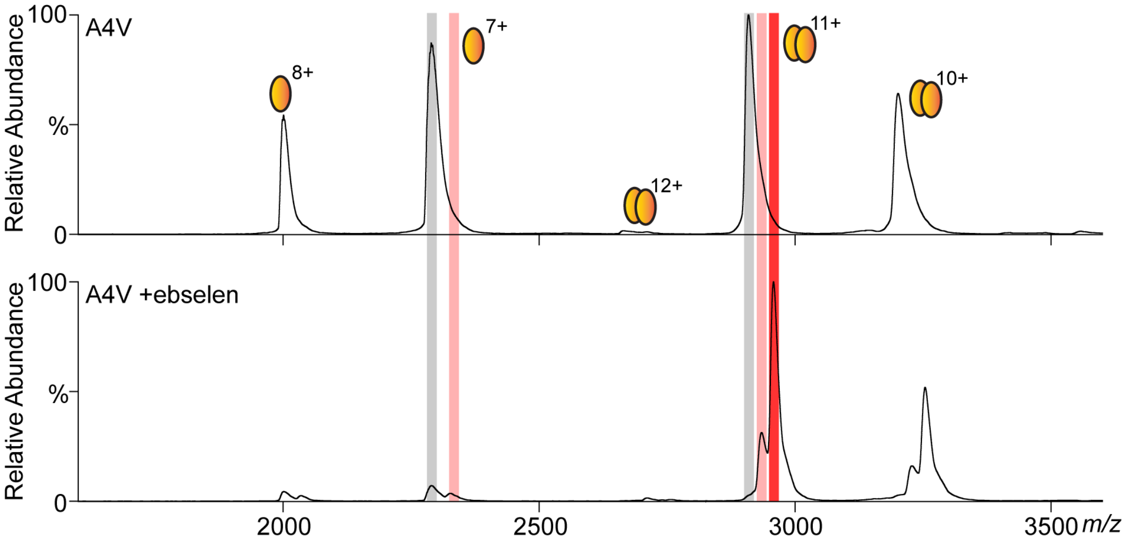

Supplementary Figure 4 | Mass spectrometry can effectively delineate differentially modified protein species within a heterogeneous mixture. Native mass spectrometry of A4V (top) and A4V^{ebselen} (bottom) at a monomer protein concentration of 1.25 µM, where ellipsoids represent oligomeric state and charge, showing the ability of mass spectrometry to separate protein species (grey = unmodified, light red = 1 x ebselen, dark red = 2 x ebselen), allowing for accurate determination of binding interactions between specific partners.

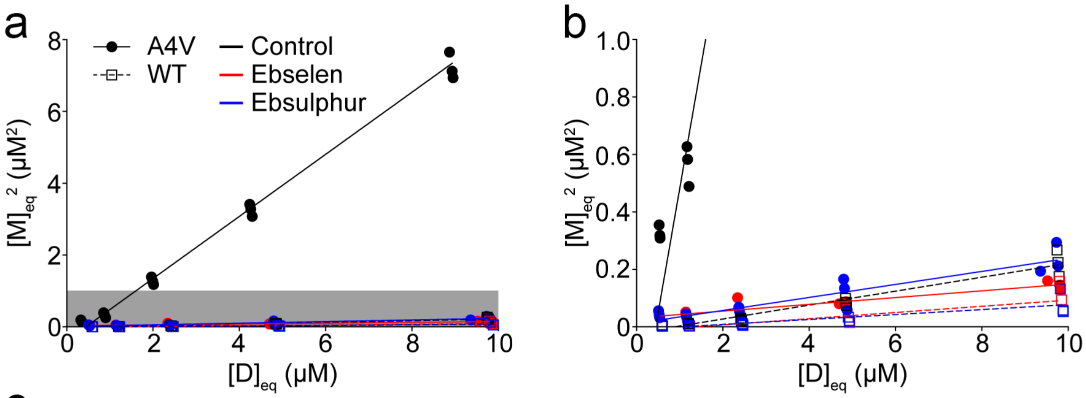

c

Mutant	Control	Ebselen	Ebsulphur
WT	0.98	0.91	0.96
A4V	0.99	0.93	0.89
H46R/H48Q	0.89	0.95	0.90
G93A	0.90	0.98	0.94
I149T	0.92	0.96	0.92

Supplementary Figure 5 | SOD1 dimer dissociation constant (K_d) determined by linear regression. Plotting the measured concentration of monomer ($[M]_{eq}^2$) against measured dimer concentration ($[D]_{eq}$) gives a linear relationship from which the gradient gives an apparent dissociation constant. (**a**) Comparing A4V to SOD1 showing control A4V has a substantially greater slope than wild-type samples and ligand-bound A4V SOD1. (**b**) Expansion of the grey region from panel (**a**) showing the similarity in slope of the ligand-bound A4V to that of wild-type control. (**c**) R values from dimer K_D determination plots.

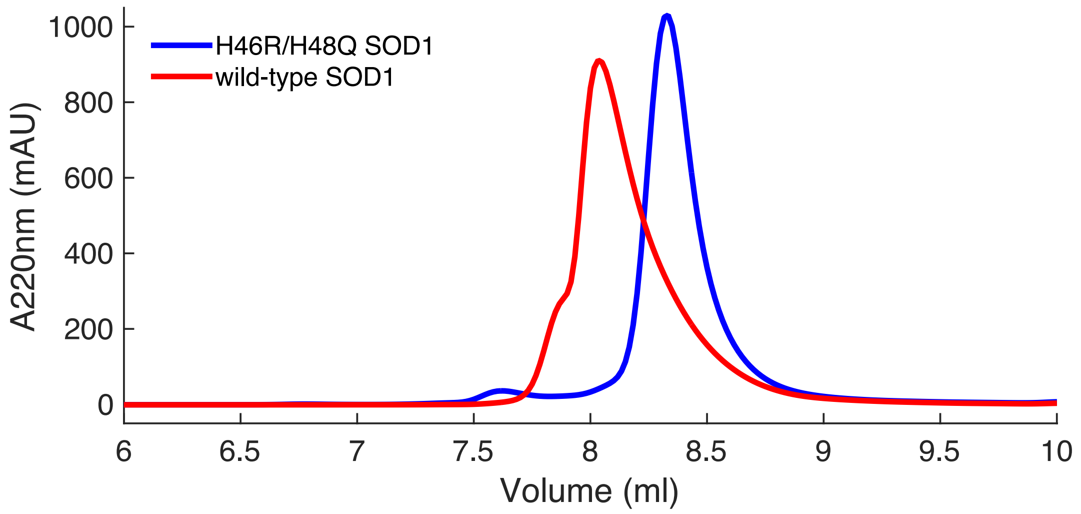

Supplementary Figure 6 | E,Zn-H46R/H48Q SOD1 is completely monomeric at low micromolar concentration. 1 nmole of H46R/H48Q SOD1 was separated by SEC and compared with the same amount of wild-type SOD1 to ascertain the oligomeric state.

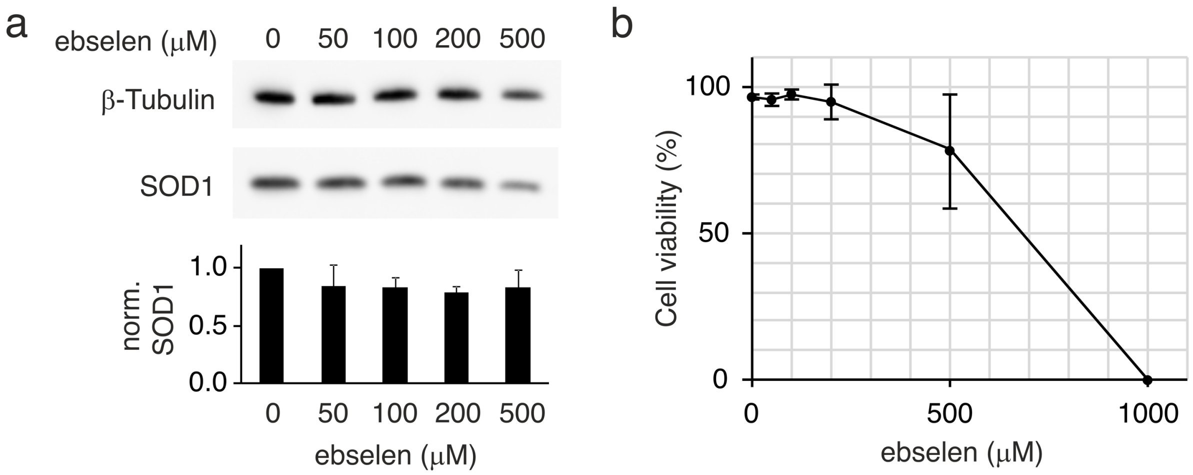

Supplementary Figure 7 | SOD1 expression levels and cell viability following ebselen treatment. (**a**) Expression levels of wild-type SOD1 in cells treated for 24 h with increasing concentrations of ebselen, normalized with respect to the SOD1 level in the untreated sample; error bars represent s.d. (n = 3). (**b**) cell viability measured by trypan blue staining as a function of ebselen concentration; error bars represent s.d. (n = 3).

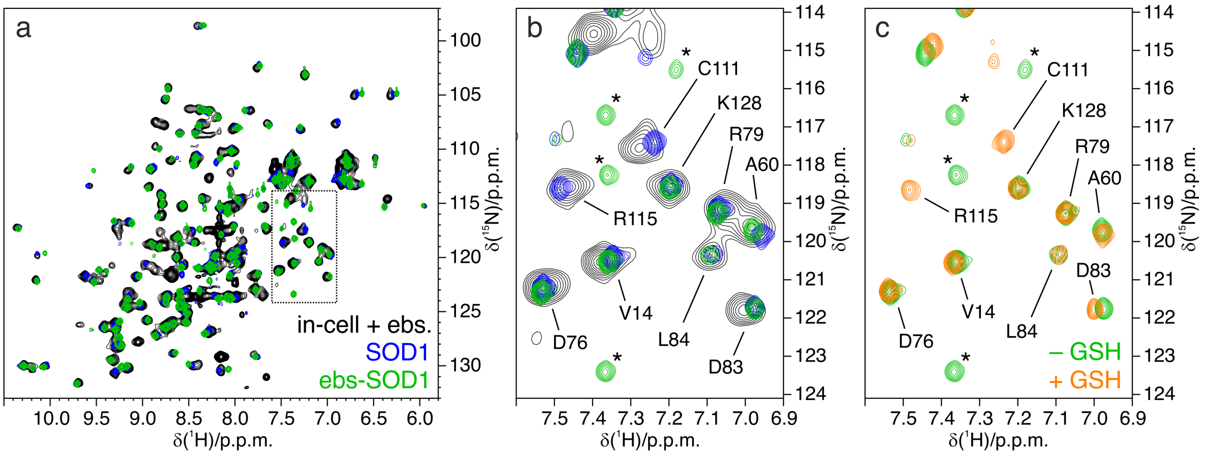

Supplementary Figure 8 | Ebselen conjugation to SOD1 Cys111 does not occur in living cells, and is reverted *in vitro* by reducing agents. (**a**) Overlay of ^{1}H-^{15}N NMR spectra acquired on human cells expressing [U-^{15}N] WT E,Zn-SOD1 in presence of ebselen in the external medium (black) and purified, disulphide-oxidized [U-^{15}N] E,Zn-SOD1 before (blue) and after (green) conjugation with ebselen. (**b**) detail of the same NMR spectra as in (**a**) showing perturbed signals arising from residues close to C111 upon conjugation with ebselen. Some signals in the ebs-SOD1 spectrum were not sequence-specific assigned and are marked with an asterisk. (**c**) Overlay of NMR spectra of purified, [U-^{15}N] ebselen-E,Zn-SOD1 in absence (green) and in presence (orange) of 1 mM glutathione (GSH); both 1 mM GSH and 1 mM dithiothreitol (data not shown) cause the complete loss of ebselen.

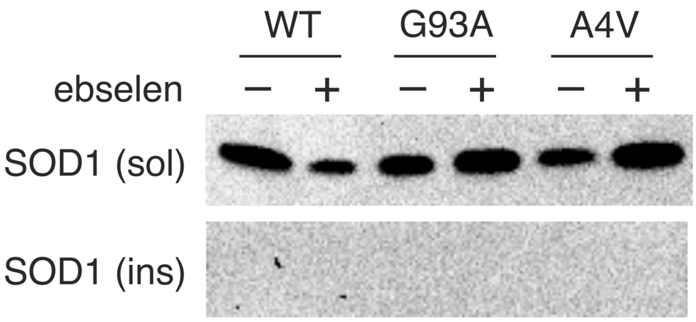

Supplementary Figure 9 | SOD1 aggregation assay. SOD1 levels assessed by Western Blot analysis on the soluble (sol) and the insoluble (ins) fractions of HEK293T cells overexpressing WT and mutant SOD1 in the absence and in the presence of ebselen. The (detergent-soluble) insoluble fraction was completely dissolved in buffer containing 0.5% Nonidet P-40 and loaded at the same dilution as the soluble fraction. SOD1 aggregates are not detected under these experimental conditions.

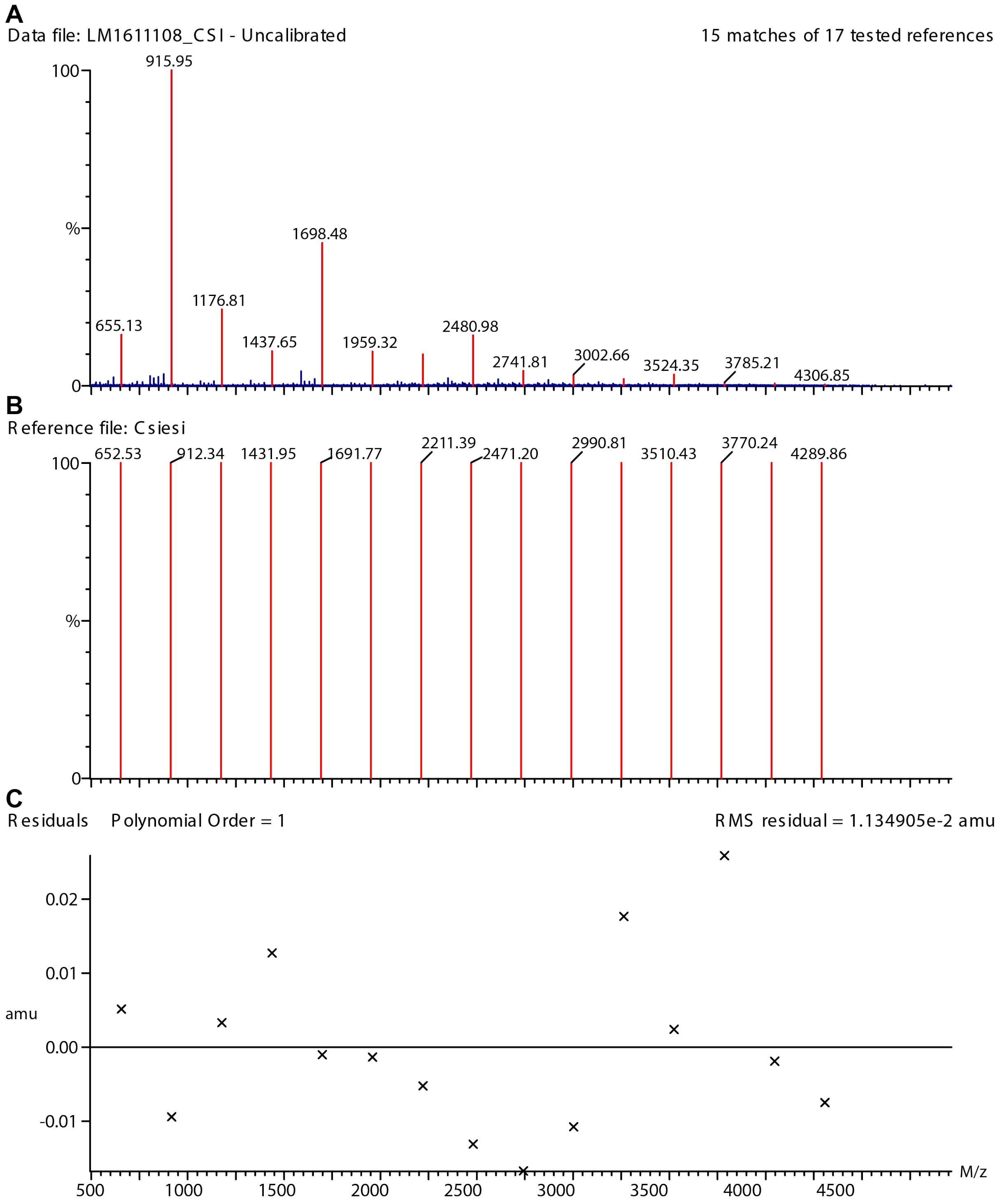

Supplementary Fig. 10 | Mass spectrometry calibration resulting from a nano-electrospray of 1 mg/ml caesium iodide in 50% n-propanol. (A) The combined and smoothed spectra of the calibration showing the major peaks in red. (B) The reference file that is used to match the peaks from panel A. (C) Mean residual plot showing the resulting error from any mass determination within the m/z range of the detected CsI clusters.

Ebselen conc.: 200 uM
T25 flasks

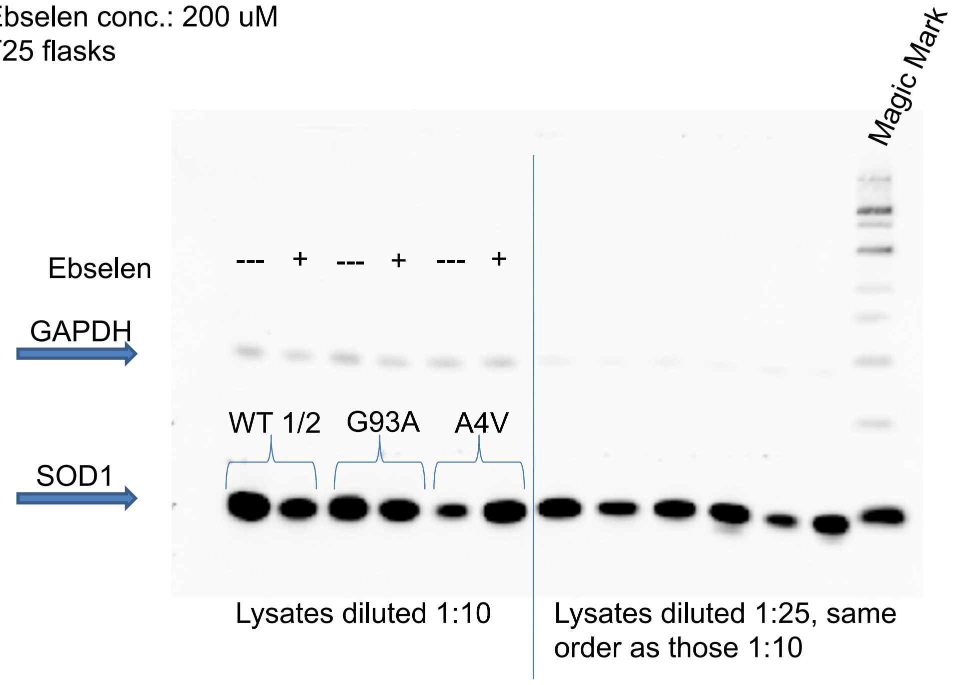

Magic Mark: 220, 120, 100,80, 60,50, 40,30, 20 kDa

Supplementary Fig. 11 | Original, uncropped western blot found in Fig. 5c.

Supplementary Table 1 | Oligonucleotide sequences

SDM oligo name	Sequence
A4V SOD1	
Sense	5′-ATGGCGACGAAGGTCGTGTGCGTGCTG-3′
Antisense	5′- CAGCACGCACACGACCTTCGTCGCCAT-3′
H46R SOD1	
Sense	5′-AGGCCTGCATGGATTCCGTGTTCATGAGTTTGGAG-3′
Antisense	5′-CTCCAAACTCATGAACACGGAATCCATGCAGGCCT-3′
H48Q (H46R background)	
Sense	5′-TGCATGGATTCCGTGTTCAAGAGTTTGGAGATAATACAG-3′
Antisense	5′-CTGTATTATCTCCAAACTCTTGAACACGGAATCCATGCA-3′
G93A SOD1	
Sense	5′-GACTGCTGACAAAGATGCTGTGGCCGATGTGTC-3′
Antisense	5′-GACACATCGGCCACAGCATCTTTGTCAGCAGTC-3′
I149T SOD1	
Sense	5′-GTCGTTTGGCTTGTGGTGTAACTGGGATCGCCC-3′
Antisense	5′-GGGCGATCCCAGTTACACCACAAGCCAAACGAC-3′
